# *Lactobacillus rhamnosus* D3189 modulates antiviral and inflammatory responses in primary nasal epithelial cells, reducing respiratory syncytial virus shedding

**DOI:** 10.3389/fcimb.2025.1625517

**Published:** 2025-07-08

**Authors:** Tejasri Yarlagadda, Jacob Stedman, Diane Maresco-Pennisi, Andrea Coleman, Anders Cervin, Kirsten Spann

**Affiliations:** ^1^ Centre for Immunology and Infection Control, School of Biomedical Sciences, Faculty of Health, Queensland University of Technology, Brisbane, QLD, Australia; ^2^ The Ear, Nose and Throat (ENT) Research Group, Centre for Clinical Research, Faculty of Health, Medicine and Behavioral Sciences, The University of Queensland, Brisbane, QLD, Australia

**Keywords:** lactobacilli, respiratory syncytial virus, nasal epithelium, innate immunity, antiviral, inflammation

## Abstract

**Introduction:**

Respiratory syncytial virus (RSV) infection in the upper respiratory tract promotes disease progression and transmission, with excessive inflammation contributing to severe lower respiratory tract involvement. This study investigates the immunomodulatory effects of *Lactobacillus rhamnosus* D3189 on viral kinetics and innate immune responses in well-differentiated nasal epithelial cells (WD-NECs).

**Methods:**

WD-NECs from healthy adult donors (N = 8) were cultured *in vitro*, treated with *L. rhamnosus* D3189, and then infected with RSV (strain RS4) 24 hours later. Viral replication and shedding were assessed via RT-qPCR and plaque assays. Cytotoxicity and epithelial integrity were evaluated using LDH release and transepithelial electrical resistance (TEER). Inflammatory and antiviral responses were investigated using multiplex immunoassays, AlphaLISA, and ELISA.

**Results:**

RSV infection induced robust viral replication and shedding, disrupted epithelial barrier integrity, and triggered the release of pro-inflammatory cytokines and type I/III interferons. *L. rhamnosus* D3189 alone did not induce cytotoxicity or inflammation. While it had no effect on viral replication, TEER, LDH release, or IFN-λ1/3 levels, D3189 significantly enhanced IFN-β production, reduced viral shedding, and attenuated RSV-induced cytokine and chemokine responses.

**Discussion:**

*L. rhamnosus* D3189 modulates the epithelial immune response to RSV, reducing inflammation and viral shedding without compromising epithelial integrity. These findings support its potential as a novel strategy to limit RSV-associated infection and transmission.

## 1 Introduction

Human respiratory syncytial virus (RSV), also known as *Orthopneumovirus hominis*, is an enveloped, negative-sense RNA virus of the *Pneumoviridae* family that primarily infects and replicates in the airway epithelium (Collins et al., 2013; Duan et al., 2024). RSV initiates infection in the upper respiratory tract (URT) and can progress to the lower respiratory tract (LRT), causing severe diseases such as bronchiolitis and pneumonia, particularly in vulnerable people (Simões et al., 2010; McLaughlin et al., 2022). While RSV infection is often mild, one-third of cases develop severe LRT disease, posing a significant threat to infants under six months, the elderly, and immunocompromised individuals (Piedimonte and Perez, 2014; McLaughlin et al., 2022).

Since the COVID-19 pandemic, RSV epidemiology has shifted, with rising case numbers across all age groups, including older children and adults who were previously less affected (Chuang et al., 2023). While RSV infection in healthy adults is typically mild, they can act as asymptomatic or mildly symptomatic carriers, contributing to virus transmission (Kaler et al., 2023). Given that RSV reinfection occurs throughout life, controlling its spread, particularly amongst high-risk populations, remains a critical public health challenge, yet effective transmission-reduction strategies are underexplored. RSV-induced inflammation plays a key role in disease progression. Viral replication in the URT can trigger an excessive immune response in susceptible individuals, leading to increased production of proinflammatory cytokines and chemokines such as IL-1β, IL-2, IL-6, IL-8, and TNF-α (Garofalo et al., 1996; Jamaluddin et al., 1998; Casola et al., 2001; Garofalo et al., 2013). This inflammation can compromise airway epithelial integrity, disrupt mucociliary clearance, and facilitate viral spread to the LRT (Newton et al., 2016). Consequently, strategies that target RSV at its initial site of infection in the URT, could help limit both transmission and disease severity.

Recent advances in RSV vaccines and therapeutics, such as monoclonal antibodies (Palivizumab, Nirsevimab) and protein subunit vaccines (Arexvy and Abrysvo), have focused on disease prevention in infants (<2 years) and older adults (>60 years), leaving a significant portion of the population unprotected (Gatt et al., 2023; Assad et al., 2024; Moreira et al., 2024; Banooni et al., 2025; Kelleher et al., 2025). While these interventions reduce severe LRT disease, they do not offer complete protection against initial URT infection (Gatt et al., 2023). This highlights the need for approaches that enhance local mucosal immunity in the URT, particularly intranasal strategies that could reduce viral shedding, lower transmission risk, and prevent LRT involvement. Strengthening URT innate immune defenses may also provide protection against other respiratory pathogens.

Recent studies, including our own, have shown that nasal administration of *Lactobacillus* species can modulate the innate immune response of airway epithelial cells (Gabryszewski et al., 2011; Tomosada et al., 2013; Percopo et al., 2015; Clua et al., 2017; Percopo et al., 2017). In murine models, priming the nasal mucosa with probiotic bacteria has been found to enhance resistance against viral infections, including RSV (Tomosada et al., 2013; Clua et al., 2017), influenza virus (Zelaya et al., 2015), and pneumonia virus of mice (Gabryszewski et al., 2011; Percopo et al., 2015; Percopo et al., 2017). Consistent with these findings, a prospective observational study reported a higher nasopharyngeal abundance of *Lactobacillus* spp. in healthy infants compared to those with RSV-associated acute respiratory infections (Rosas-Salazar et al., 2016). Additionally, an overrepresentation of *Lactobacillus* spp. in the respiratory tract of healthy infants has been linked to a reduced risk of developing RSV-associated childhood wheezing illnesses (Rosas-Salazar et al., 2018). Among the *Lactobacillus* species with immunomodulatory properties, *Lactobacillus rhamnosus* strains show promise in improving outcomes of respiratory viral infections (Kumova et al., 2019; Yarlagadda et al., 2024). In our work, we identified a novel strain, *L. rhamnosus* D3189, which significantly reduced the release of rhinovirus (RV)-induced inflammatory markers in an *in vitro* nasal epithelium model (Yarlagadda et al., 2024), highlighting its potential to mitigate virus-induced upper respiratory tract disease by modulating inflammation.

These findings suggest that lactobacilli, particularly *L. rhamnosus* strains, may beneficially modulate the respiratory innate immune response against RSV. Building on this and previous studies, we investigated whether *L. rhamnosus* D3189 influences viral kinetics and the production of cytokines and chemokines in nasal epithelial cells following RSV challenge.

## 2 Materials and methods

### 2.1 Human primary nasal epithelial cells

This study was conducted in accordance with the Queensland University of Technology Human Research Ethics Committee (2021000292). Nasal epithelial cells (NECs) were collected from the inferior turbinates of eight healthy adult donors (6 females, 2 males, 28 ± 8.8 years old) in May 2024 using a sterile nasal mucosal curette (Arlington Scientific, USA) with informed consent. NEC cultures were established and expanded as submerged monolayers as previous described (Kicic et al., 2010; Spann et al., 2014; Kicic et al., 2016). Cells were stored in aliquots in freezing media (fetal bovine serum with 10% dimethyl sulfoxide) at passage 1 or 2 in liquid nitrogen until use.

### 2.2 Air liquid interface culture

Primary NECs were seeded (5 × 10^5^ cells/6.5 mm transwell, 0.4 μm pores; Corning Costar, USA) and cultured in PneumaCult™-Ex Plus Medium (Stemcell Technologies, Canada), as described previously (Yarlagadda et al., 2024). Upon confluence, cells were air-lifted and maintained in PneumaCult™-ALI Medium (Zhu et al., 2022; Yarlagadda et al., 2024). Cultures were differentiated for ≥3 weeks until ciliation, mucus production, and transepithelial electrical resistance (TEER) >250 Ω·cm², measured using an EVOM^2^ Epithelial Voltohmmeter (World Precision Instruments, USA), were observed. Medium was changed thrice weekly. Supplements were removed 24 h in advance of *L. rhamnosus* exposure (Yarlagadda et al., 2024).

### 2.3 *Lactobacillus rhamnosus* preparation

*L. rhamnosus* strain D3189, isolated from the URT of healthy children (Coleman et al., 2021), was selected for its *in vitro* inhibition of *Streptococcus pneumoniae*, *Haemophilus influenzae* and *Moraxella catarrhalis* (Coleman et al., 2022) and its ability to differentially modulate RV-induced innate immune responses in primary NECs from healthy adults (Yarlagadda et al., 2024). D3189 was prepared as previously described (Yarlagadda et al., 2024), grown until the end of the lag phase, and harvested for WD-NEC exposure (Yarlagadda et al., 2024).

### 2.4 Virus propagation

A clinical isolate of RSV (RS4), generously provided by Professor Paul Young (University of Queensland), was used in this study. RSV was propagated in human epithelial type 2 (HEp-2) cells (ATCC^®^ CCL-23™) and purified as described previously (Spann et al., 2014). Virus stocks were quantified using an immuno-plaque assay (Spann et al., 2014), which was also used to quantify viral shedding in apical washes from experimental NEC cultures.

Briefly, virus stocks were serially diluted 10-fold and used to infect confluent monolayers of HEp-2 cells in duplicate. Two hours post-infection, the cells were overlaid with 0.8% methyl cellulose in OptiMEM/2% fetal bovine serum/1% antibiotic-antimycotic (Thermo Fisher Scientific, USA), and incubated at 37°C with 5% CO_2_ for 7 days. Cells were then fixed with 60% methanol/40% acetone and blocked with 5% skim milk powder in PBS. RSV plaques were detected using a goat anti-RSV polyclonal antibody (1:500; Sigma-Aldrich, USA), followed by a horseradish peroxidase (HRP)-conjugated anti-goat secondary antibody (1:500; Invitrogen, USA). Both antibody incubations were carried out for 2 hours at 37°C with 5% CO_2_. Plaques were visualized using a peroxidase substrate with metal enhancer (SigmaFast DAB; Sigma-Aldrich) and counted to calculate viral titers as plaque-forming units (PFU) mL^-1^ (Yarlagadda et al., 2024).

### 2.5 Probiotic exposure, viral infection and sample analysis

Duplicate cultures of NECs were exposed to *L. rhamnosus* D3189 either alone or prior to RSV infection, as described previously (Yarlagadda et al., 2024). Replicate cultures were treated with RSV alone or cell culture media as mock-infection controls. For probiotic pre-treatment, NECs were apically exposed to 80 µL of cell culture media containing either D3189 (2.5 × 10^7^ CFU mL^-1^) or media alone (virus-only control) for 4 h at 37°C with 5% CO2. Following this exposure, the inoculum was removed to re-establish the air-liquid interface, and the cells were incubated for an additional 20 h. After this incubation period, NECs were either apically treated with 80 µL of cell culture media alone (bacteria-only control) or infected with 80 µL of RSV (7.5 × 10^6^ PFU mL^-1^) for 4 h at 37°C with 5% CO_2_. The viral inoculum was then removed, and cultures were maintained at the air-liquid interface for an additional 3 days. Duplicate cultures of NECs from 5 donors were exposed to *L. rhamnosus* GG (LGG) either alone or prior to RSV infection as above, as a comparison to D3189.

At 3 days post-infection, TEER was measured to assess epithelial barrier integrity. Apical washes (200 µL PBS) were used to quantify shed virus via immuno-plaque assays (Spann et al., 2014), while both apical wash and basal media were analyzed for cytokine and chemokine release using AlphaLISA, ELISA, or magnetic bead assays. Bacterial carriage in the apical wash was quantified using the drop plate technique to determine CFU mL-1. Finally, cells on transwell membranes were lysed in 150 μL of TRIzol^®^ reagent (Thermo Fisher Scientific) for total RNA extraction and reverse transcription quantitative PCR (RT-qPCR).

### 2.6 Viral gene expression analysis by two-step RT-qPCR

Total RNA was extracted from cell lysates using TRIzol/chloroform phase separation method (Spann et al., 2004), followed by purification and concentration with the PureLink™ RNA Mini Kit (Thermo Fisher Scientific) according to the manufacturer’s instructions. RNA quality and quantity were assessed using a Nanodrop Lite Spectrophotometer (Thermo Fisher Scientific). cDNA was synthesised from 200 ng of total RNA using the SuperScript™ III First-Strand Synthesis SuperMix (Invitrogen, USA) with Oligo(dT)_20_ primers, following the manufacturer’s protocol. RSV nucleocapsid (N) mRNA was quantified using the QuantiNova SYBR Green PCR Kit (Qiagen) and RSV N-specific primers (Forward 5’ – 3’: AAG GGA TTT TTG CAG GAT TGT TT, Reverse 5’ – 3’: CTC CCC ACC GTA GCA TTA CTT G). qPCR was performed on the QIAGEN Rotor-gene-Q according to the following cycling conditions: 5˚C for 5 mins, 45 cycles of 95˚C for 5 seconds and 60˚C for 10 seconds. Gene expression was normalized to β-actin mRNA (Forward 5’ – 3’: TAC GCC AAC ACA GTG CTG TCT, Reverse 5’ – 3’: TCT GCA TCC TGT CGG CAA T) (Yarlagadda et al., 2024).

### 2.7 LDH activity

As an index of cytotoxicity, lactate dehydrogenase (LDH) release was measured using basal media collected 3 days post infection (p.i.) with RSV, using the LDH-Glo Cytotoxicity Assay Kit (Promega) as per the manufacturer’s instructions. The luminescence of the samples was recorded using a CLARIOstar Omega reader. Enzyme activity was expressed as mU/mL.

### 2.8 Cytokine and chemokine quantification of culture supernatants

Apical wash and basal media were used to quantify IFN-β and IFN-λ1/3 production using an AlphaLISA (Perkin Elmer) and the R&D Systems Human DuoSet ELISA kit, respectively, according to each manufacturer’s instructions. Concentration for apical and basal compartments were combined for total protein production for each individual culture. Inflammatory markers (G-CSF, GM-CSF, IFN-γ, IL-1β, IL-2, IL-4, IL-5, IL-6, IL-7, IL-8, IL-10, IL-12, IL-13, IL-17, MCP-1, MIP-1β and TNF-α) secreted in the basal media were quantified using the Bio-Plex Pro Human Cytokine 17-plex Panel (Bio-Rad, USA) on the Bio-Plex^®^-200 system and concentrations were determined using Bio-Plex Manager Software.

### 2.9 Statistical analysis

Statistical analyses were conducted using the GraphPad Prism 10.4.1 software. Paired *t*-tests (Wilcoxon signed-rank test) and one-way ANOVAs (Friedman test and uncorrected Dunn’s *post hoc* test) were used to assess differences between treatment groups. Statistical significance was defined as *p* < 0.05 with a 95% confidence interval.

## 3 Results

### 3.1 *L. rhamnosus* D3189 reduced RSV shedding, although not intracellular transcription

**Figure 1:**
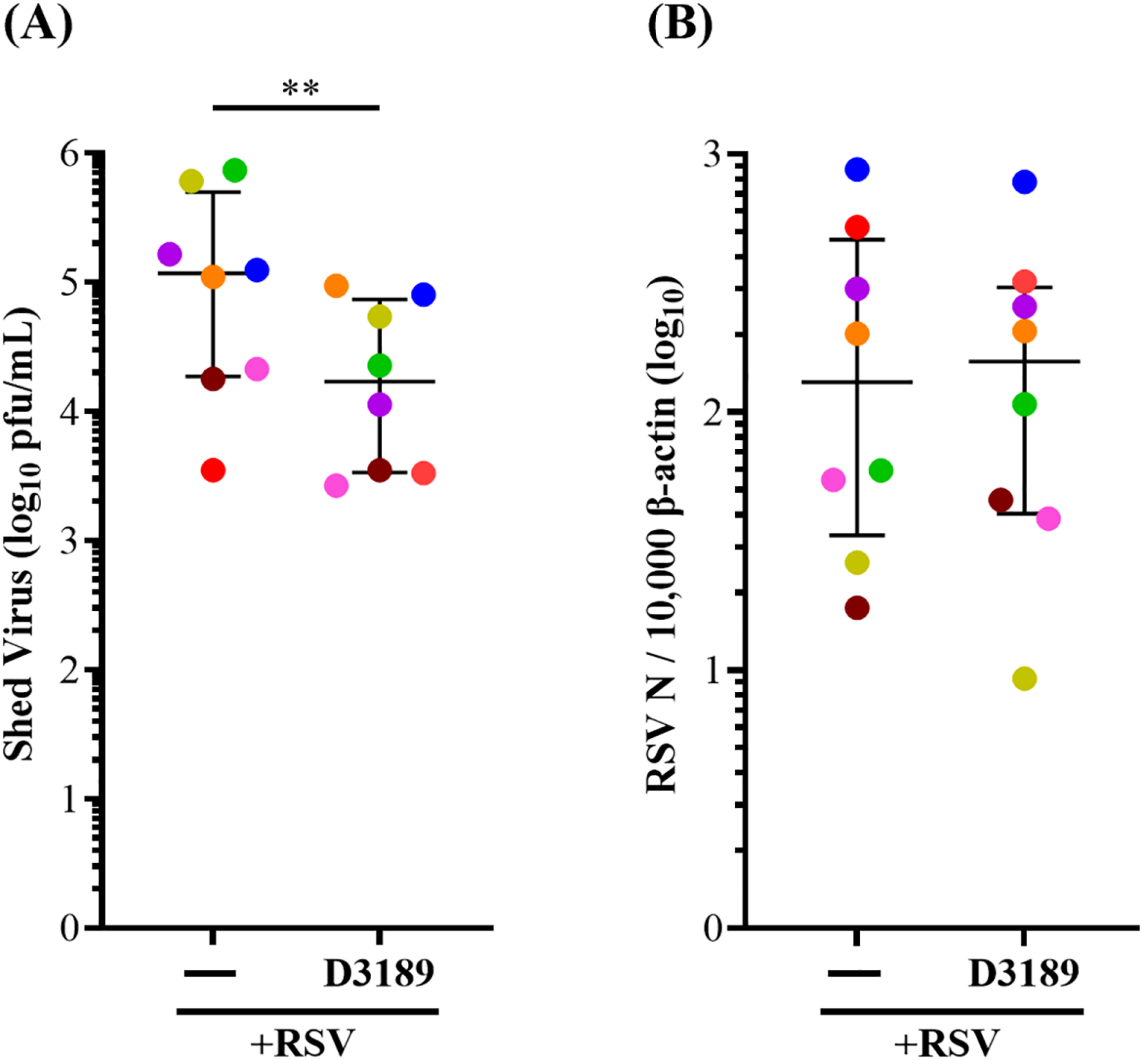
Effect of *L. rhamnosus* D3189 on RSV load 3 days post-infection. WD-NECs were pretreated with D3189 (80 µL of 2.5 × 10^7^ CFU mL^-1^) or media (negative control) for 24 h before RSV infection (80 µL of 7.5 × 10^6^ PFU mL^-1^) or mock exposure (virus-negative control). At 3 days post-infection, apical washes were collected. **(A)** Shed virus was quantified by immuno-plaque assay. **(B)** Viral transcription was assessed by RT-qPCR using RSV N-specific primers, normalised to β-actin expression. Data are presented as median and interquartile range, analysed by Wilcoxon signed-rank test. Each data point represents the mean of duplicate cultures per donor, with donors distinguished by colour (*n* = 8). **, *p* < 0.01.

To assess the effect of *L. rhamnosus* D3189 on viral kinetics, we measured shed virus in apical washes and RSV N mRNA expression at 3 days p.i. Pretreatment with D3189 significantly reduced viral shedding (**Figure 1A**), suggesting a potential impact on viral release or egress. However, there was no significant change in N gene mRNA levels (**Figure 1B**) indicating no effect on viral transcription.

Bacterial carriage was evaluated four days post-exposure to assess potential variations in lactobacilli levels among donors at the time of sampling and to confirm colonization by D3189. All cultures exhibited comparable bacterial loads in the apical wash, measured as CFU mL^-1^, confirming successful colonization (**Supplementary Figure 1**).

### 3.2 *L. rhamnosus* D3189 does not alter cytotoxicity or barrier integrity during RSV infection

**Figure 2:**
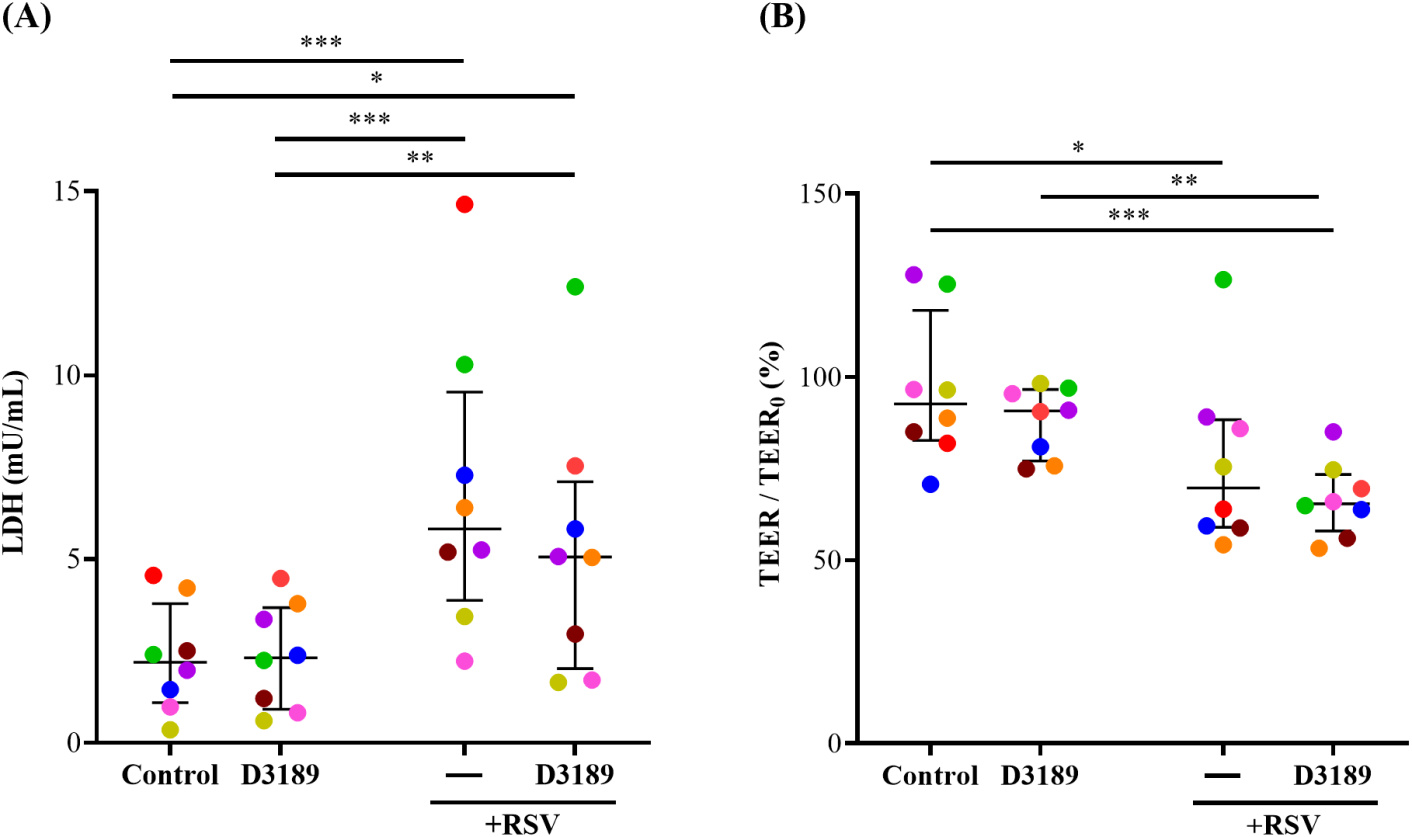
Effect of *L. rhamnosus* D3189 on LDH release and TEER 3 days post-infection with RSV. WD-NECs were pretreated with D3189 (80 µL of 2.5 × 10^7^ CFU mL^-1^) or media (negative control) for 24 h before RSV infection (80 µL of 7.5 × 10^6^ PFU mL^-1^) or mock exposure (virus-negative control). At 3 days post-infection, **(A)** LDH release in the basal media was quantified by LDH-Glo Cytotoxicity Assay Kit, and **(B)** TEER was measured using the EVOM^2^ Epithelial Voltohmmeter and is presented as a percentage fold change relative to baseline TEER. Data are presented as median and interquartile range, analysed by the Friedman test and uncorrected Dunn’s *post hoc* test. Each data point represents the mean of duplicate cultures per donor, with donors distinguished by colour (*n* = 8). *, *p* < 0.05; **, *p* < 0.01; ***, *p* < 0.005.

LDH, a cytosolic enzyme released upon damage to the plasma membrane, was measured as an indicator of cytotoxicity. Although LDH release varied among donors, D3189 alone did not induce cytotoxicity, consistent with our previous findings (Yarlagadda et al., 2024). However, RSV infection with and without D3189 pretreatment significantly elevated LDH release compared to the uninfected or D3189 only controls, indicating that D3189 did not mitigate RSV-induced cytotoxicity (**Figure 2A**). Similarly, TEER, an indicator of tight junction integrity, was unaffected by D3189 alone but significantly reduced by RSV infection, regardless of probiotic pretreatment, suggesting that D3189 did not protect against virus-induced barrier disruption (**Figure 2B**).

### 3.3 *L. rhamnosus* D3189 does not significantly modulate the IFN response to RSV infection

**Figure 3:**
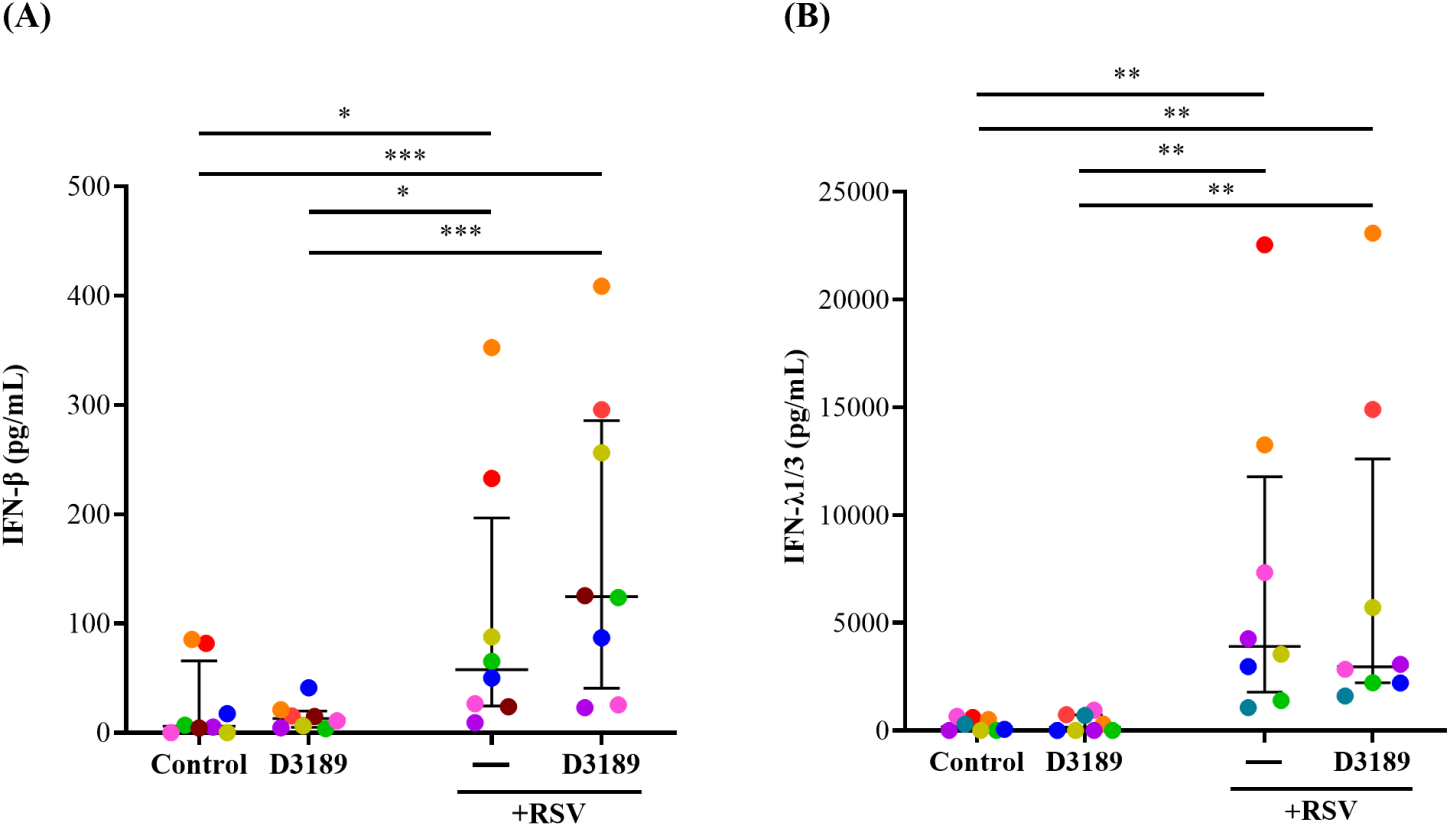
Effect of *L. rhamnosus* D3189 IFN-β and IFN-λ1/3–3 days post-infection with RSV. WD-NECs were pretreated with D3189 (80 µL of 2.5 × 10^7^ CFU mL^-1^) or media (negative control) for 24 h before RSV infection (80 µL of 7.5 × 10^6^ PFU mL^-1^) or mock exposure (virus-negative control). At 3 days post-infection, secreted **(A)** IFN-β and **(B)** IFN-λ1/3 were quantified by AlphaLISA and standard ELISA, respectively, using both apical wash and basal media. Data are presented as median and interquartile range, analysed by the Friedman test and uncorrected Dunn’s *post hoc* test. Each data point represents the mean of duplicate cultures per donor, with donors distinguished by colour (*n* = 8). *, *p* < 0.05; **, *p* < 0.01; ***, *p* < 0.005.

IFN-β and -λ are principal antiviral proteins induced by RSV infection of airway epithelial cells (AECs) (Bergeron et al., 2023). Given the observed reduction in viral shedding, we investigated whether D3189 influenced the antiviral response by enhancing IFN production. IFN-β and -λ1/3 release were quantified in apical washes and basal media from duplicate cultures and combined to determine secretion. D3189 alone did not induce IFN release by WD-NECs (**Figures 3A**, **B**). As expected, RSV infection significantly increased IFN-β and IFN-λ1/3 production compared to uninfected controls, although with large donor variability. While D3189 pretreatment did not statistically significantly alter this response, there was a strong trend toward increased IFN-β levels in co-exposed cultures (**Figure 3A**). Notably, 6 out of 8 donors (orange, red, yellow, green, brown, blue) exhibited higher IFN-β secretion when pretreated with D3189 compared to no pretreatment, suggesting donor specific variability regarding the effect on RSV-induced IFN-β production.

### 3.4 *L. rhamnosus* D3189 attenuates RSV-induced inflammatory responses

**Figure 4:**
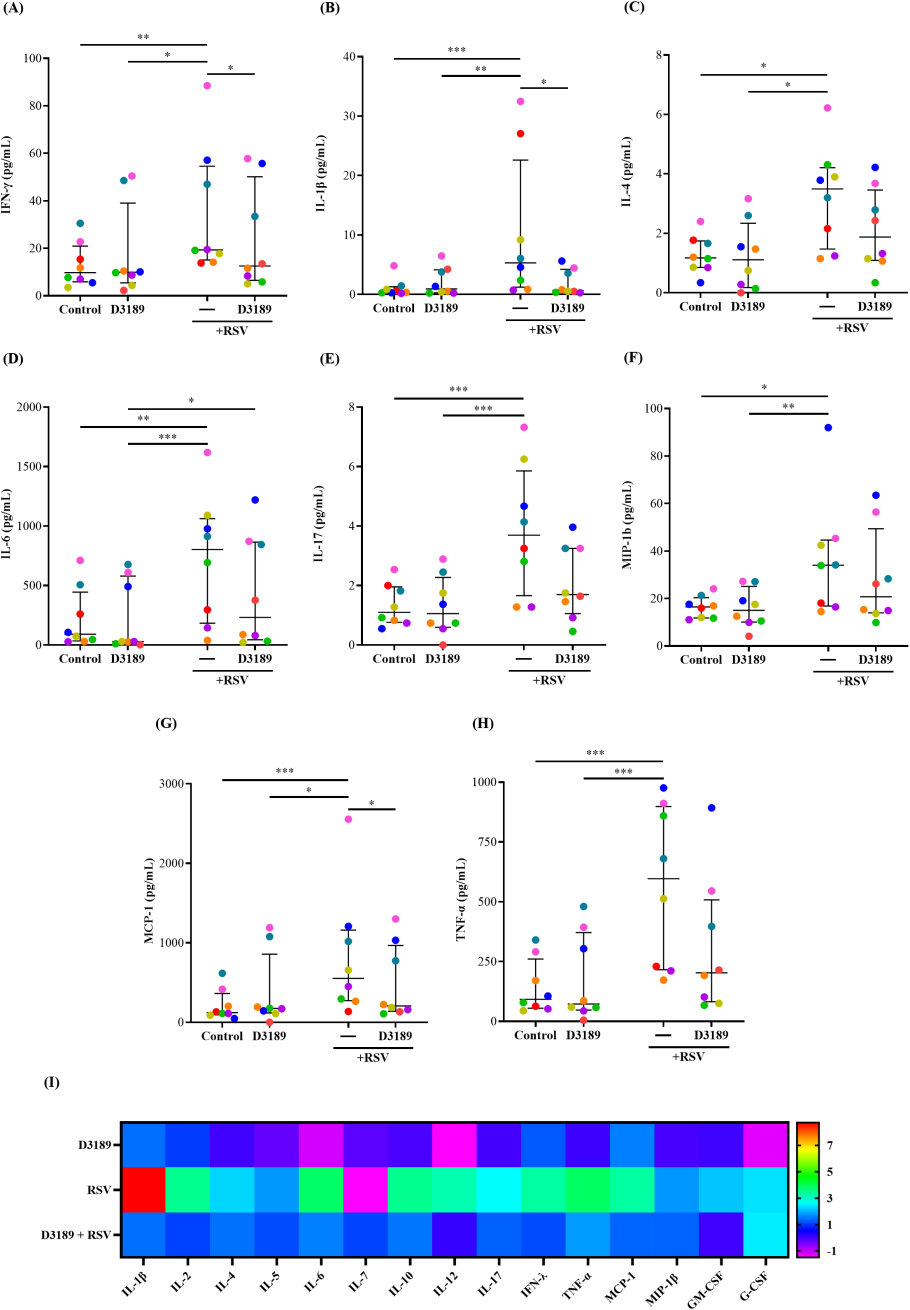
Effect of *L. rhamnosus* D3189 pro-inflammatory mediator release 3 days post-infection with RSV. WD-NECs were pretreated with D3189 (80 µL of 2.5 × 10^7^ CFU mL^-1^) or media (negative control) for 24 h before RSV infection (80 µL of 7.5 × 10^6^ PFU mL^-1^) or mock exposure (virus-negative control). At 3 days post-infection, secreted cytokines and chemokines were quantified in basal media using the Bio-plex pro human cytokine 17-plex assay. Data points represent secreted **(A)** IFN-γ, **(B)** IL-1β, **(C)** IL-4, **(D)** IL-6, **(E)** IL-17, **(F)** MCP-1, **(G)** MIP-1β and **(H)** TNF-α from each donor. A **(I)** heatmap illustrates fold changes in cytokine and chemokine production relative to uninfected controls for all analytes detected with the assay’s limit of detection. Data are presented as median and interquartile range, analysed by the Friedman test and uncorrected Dunn’s *post hoc* test. Each data point represents the mean of duplicate cultures per donor, with donors distinguished by colour (*n* = 8). *, *p* < 0.05; **, *p* < 0.01; ***, *p* < 0.005.

Host innate inflammatory responses to RSV infection play a critical role in determining disease severity, as excessive inflammation, characterized by increased production of proinflammatory cytokines and chemokines have been linked to more severe RSV disease, prolonged illness, and heightened risk of secondary infections (Sun and López, 2017; Agac et al., 2023). We investigated whether D3189 could modulate the release of proinflammatory cytokines and chemokines from RSV-infected WD-NECs, particularly to assess whether pretreatment dampens inflammation, as we previously observed in RV-infected WD-NECs (Yarlagadda et al., 2024). A 17-plex bead array was performed using basal medium to quantify key proinflammatory cytokines and chemokines. As expected, RSV infection significantly induced the release of IFN-γ, IL-1β, IL-4, IL-6, IL-17, MCP-1, MIP-1β and TNF-α compared to uninfected controls (**Figures 4A–H**). D3189 alone did not induce cytokine or chemokine expression, consistent with our previous findings (Yarlagadda et al., 2024). However, D3189 pretreatment prior to RSV infection significantly attenuated IFN-γ, IL-1β and MCP-1 production (**Figures 4A, B, G**, respectively), with a strong trend toward reduced IL-17 and TNF-α levels (**Figures 4E, H**, respectively). Notably, this dampening effect was most pronounced in donors exhibiting the highest inflammatory responses to RSV infection (as indicated by color coding). A heatmap (**Figure 4I**) illustrating fold changes over uninfected controls further demonstrated an overall reduction in inflammatory cytokine and chemokine release following D3189 pretreatment, although not all changes reached statistical significance. These findings suggest that D3189 has the potential to suppress RSV-induced inflammation in WD-NECs.

## 4 Discussion

RSV is a major etiological agent of respiratory infections and acute respiratory disease, contributing significantly to morbidity and mortality rates worldwide (Li et al., 2022). Despite extensive research efforts, both prophylactic and therapeutic strategies remain inadequate in reducing infection in the URT. Our previous work demonstrated that pretreatment with specific *L. rhamnosus* strains reduced RV-induced cytokine and chemokine release in WD-NECs without affecting viral load (Yarlagadda et al., 2024). Building on these findings, we hypothesized that D3189 might confer protection against RSV infection in the same WD-NEC model. Our results reveal that pretreatment with D3189 reduces viral shedding and significantly attenuates pro-inflammatory cytokine and chemokine release following RSV infection. To our knowledge, this is the first study to report the immunomodulatory effects of lactobacilli against RSV infection in primary WD-NECs.

Studies in murine models of IAV, RSV, and pneuomovirus of mice (PVM) infection have demonstrated that intranasal administration of lactobacilli reduces viral load in the respiratory tract and improves survival outcomes (Hori et al., 2001; Gabryszewski et al., 2011; Youn et al., 2012; Park et al., 2013; Tomosada et al., 2013). Although our model of WD-NECs lacks a functional immune system we did also observe reduced RSV viral shedding correlated to pre-exposure to *L. rhamnosus* D3189. Interestingly, this effect was not observed in our previous study with RV-infected WD-NECs, where D3189 had no impact on viral load (Yarlagadda et al., 2024), indicating virus-specific interactions with D3189. RSV infects AECs by attaching to surface receptors and entering via membrane fusion (Battles and McLellan, 2019). Once inside, the viral RNA-dependent RNA polymerase transcribes genes in a sequential 3’ to 5’ gradient. Full-length genome replication and synthesis of viral proteins support virion assembly and apical release through budding (Shang et al., 2021). This life cycle underpins RSV’s pathogenicity, especially in infants, where epithelial injury and exaggerated inflammatory responses contribute to bronchiolitis. In our study, the observed reduction in RSV shedding without a corresponding decrease in intracellular N gene mRNA suggests that D3189 may interfere with later stages of the virus life cycle, such as assembly, budding, or egress rather than transcription or genome replication. However, the precise mechanisms by which D3189 exerts these effects remain to be elucidated.

We hypothesized that the reduction in RSV virus shedding by pre-exposure to D3189 may correlate with enhanced production of key antiviral cytokines, specifically type I (IFN-α/β) and type III (IFN-λ) interferons, which are critical in the innate antiviral immune response and viral clearance (Yang and Li, 2020). Mouse and airway cell studies have shown that lactobacilli enhance resistance to viral infections, including RSV, by upregulating IFN-β production, which subsequently boosts the expression of viral-sensing receptors such as TLR3 and DDX58, and antiviral factors including Mx1 and OAS1, which limit viral replication (Tomosada et al., 2013; Islam et al., 2021). In our WD-NEC study D3189 alone did not induce either IFN-β or -λ production (Yarlagadda et al., 2025), although similarly to *L. rhamnosus*-treated mice challenged with RSV (Tomosada et al., 2013), there was a trend for virus-induced IFN-β to be further enhanced by D3189 exposure, at least for 6 of the 8 donors. The boost in IFN may have partially contributed to reduced infectious virus shedding, as RSV both induces IFN-β and is susceptible to IFN-β for viral clearance (Hijano et al., 2019). Unlike RSV, in our previous study using WD-NECs we did not observe any induction of IFN-β by RV, or enhanced IFN-β induction in combination with D3189 exposure. This difference can be attributed to the fact that RV does not rely on IFN-β for viral clearance, and is a poor inducer of IFN-β, unlike RSV (Mueller and Rouse, 2008; Yuan et al., 2022).

To assess whether D3189 modulates RSV-induced cytotoxicity, we measured LDH release as an indicator of epithelial damage. D3189 alone had no deleterious effect on WD-NECs, consistent with our previous findings (Yarlagadda et al., 2024). However, while our earlier study assessed D3189 exposure over 48 hours, the present study extended this duration to four days post-exposure, further confirming the absence of cytotoxic effects associated with prolonged D3189 exposure. As anticipated, RSV induced LDH release, consistent with multiple studies reporting LDH detection in nasopharyngeal samples as a marker of RSV-induced epithelial injury and a predictor of disease severity (Vázquez et al., 2019). Previous studies in mice have demonstrated that nasal delivery of lactobacilli prior to RSV infection or poly(I:C) challenge significantly reduces LDH release, suggesting a protective effect against virus-induced cytotoxicity (Tomosada et al., 2013). However, despite the significant reduction in viral shedding, D3189 pretreatment did not mitigate RSV-induced LDH release within the 3 days of experimentation. This finding suggests that, unlike the mouse studies, D3189 does not confer similar protection in the context of RSV infection in the WD-NEC model. These results align with our previous study where D3189 and other *L. rhamnosus* isolates had no effect on RV-induced LDH release (Yarlagadda et al., 2024). The mechanisms underlying this response remain unclear, however they may be influenced by distinct host immune interactions, where factors essential for lactobacilli-enhanced epithelial repair are absent from the WD-NEC model. Further investigations are warranted to delineate the precise factors governing lactobacilli-mediated cytoprotective effects in viral infections.

RSV infection is known to compromise airway epithelial barrier integrity, leading to increased permeability and disruption of tight junctions (Kast et al., 2017; Smallcombe et al., 2019). While the impact of probiotics on epithelial barrier dysfunction in the URT remains largely unexplored, some evidence suggests that lactobacilli can enhance and regulate epithelial barrier function in AECs (De Boeck et al., 2020; De Rudder et al., 2020). In the present study, exposure to D3189 did not compromise tight junction integrity, indicating an absence of deleterious effects. As anticipated, RSV infection led to a significant reduction in TEER, consistent with previous reports demonstrating RSV-mediated disruption of AEC tight junctions (Kast et al., 2017; Smallcombe et al., 2019). However, similarly to LDH release, this barrier dysfunction was not modulated by D3189 pretreatment, suggesting that D3189 does not confer protective effects on tight junction integrity under these conditions.

RSV infection triggers a robust cytokine and chemokine response essential for viral clearance. However, it is often associated with a dysregulated hyper-inflammatory response, particularly in RSV bronchiolitis (Das et al., 2005; Rayavara et al., 2018). RSV is a potent activator of NF-κB signaling pathways in airway epithelial cells, leading to upregulation of inflammatory mediators such as IL-1α/β, IL-6, IL-8, TNF-α, IFN-γ, IL-17, MCP-1, MIP-1α/β, RANTES, and IP-10 (Garofalo et al., 1996; Jamaluddin et al., 1998; Casola et al., 2001; Garofalo et al., 2013). Consistent with this, we observed that RSV infection induced the release of IFN-γ, IL-1β, IL-4, IL-6, IL-17, MCP-1, MIP-1β, and TNF-α from WD-NECs, all of which influence RSV pathogenesis and disease severity. Pre-exposure to D3189 significantly reduced RSV-induced release of IFN-γ, IL-1β, and MCP-1, with additional trends towards reduced IL-17 and TNF-α. While IFN-γ is essential for antiviral immunity, excessive levels contribute to airway obstruction and inflammation (Estripeaut et al., 2008). IL-1β induces epithelial damage and mucus hypersecretion, while IL-17 contributes to neutrophilic damage and post-viral wheezing (Mukherjee et al., 2011). MCP-1 recruits monocytes, intensifying inflammation and viral spread, while TNF-α enhances epithelial apoptosis and mucus production, further compromising airway function (Bergeron and Tripp, 2021). The observed shift in cytokine and chemokine profiles following D3189 treatment suggests an immunomodulatory effect that could mitigate RSV-induced inflammation and epithelial damage. We previously hypothesized that *L. rhamnosus* D3189 metabolites might inhibit NF-κB signaling to suppress pro-inflammatory cytokine and chemokine production (Yarlagadda et al., 2024). In this study, D3189 may have directly dampened cytokine and chemokine production as we have observed previously (Yarlagadda et al., 2024), although the reduction in virus shedding with D3189 pretreatment may have also indirectly influenced reduced immune activation thereby contributing to lower inflammatory mediator release. Thus, D3189 appears to both directly modulate immune responses and indirectly reduce inflammation by limiting viral shedding, alleviating RSV-associated immune activation.

We previously observed that different *L. rhamnosus* strains modulate host responses to RV infection in WD-NECs differently (Yarlagadda et al., 2024). To determine whether the observed antiviral effects were unique to *L. rhamnosus* D3189, we also tested another commonly studied probiotic strain, LGG, in a subset of donors (n = 5). These results, presented in **Supplementary Figure 2**, showed that LGG did not significantly reduce RSV shedding, enhance IFN-β expression, or attenuate RSV-induced proinflammatory responses. Moreover, LGG co-exposure resulted in greater disruption of epithelial barrier integrity, as reflected by reduced TEER compared to RSV infection alone. These findings suggest that the antiviral and immunomodulatory effects observed in this study are strain-specific and highlight the importance of functional screening when selecting probiotic candidates for respiratory applications.

Although this study offers valuable insights, several limitations should be considered when translating the results of probiotic therapy from the *in vitro* model to clinical settings. The WD-NEC model lacks immune cell components and other features of a fully functional mucosa, limiting our observations to the responses of structural cells to both RSV infection and probiotic exposure. Additionally, variability in human donor responses, driven by genetic and environmental differences, may influence NEC responses. Expanding the donor cohort in future studies may help clarify the broach applicability of the observed effects.

To build on findings of this study, future research will focus on elucidating the mechanisms underlying the antiviral effects of *L. rhamnosus* D3189. Specifically, we will investigate whether reduced RSV shedding results from inhibited release of fully assembled and infectious virions from NECs. To determine whether bacterial viability is required, we will compare the effects of live and heat-inactivated bacteria as well as conditioned media, to understand whether secreted factors or direct surface interactions are responsible. Time-course experiments introducing D3189 at different stages of infection will further define the window during which the probiotic is effective. Additionally, we will investigate D3189’s immunomodulatory activity by using inhibition studies to clarify host pathways involved in antiviral defense.

In conclusion, our study highlights the potential of *L. rhamnosus* D3189 as a promising immunomodulatory agent in the context of RSV infection. D3189 enhances IFN-β production, reduces viral shedding, and mitigates the release of pro-inflammatory cytokines and chemokines, which could collectively reduce the severity of RSV-induced inflammation within the URT. Reduced viral shedding may also indicate a positive influence of nasal probiotics in reducing the risk of transmission. However, detailed mechanistic studies are required to fully understand how D3189 mediates these effects and to inform its optimal use in clinical applications.

## Data Availability

The raw data supporting the conclusions of this article will be made available by the authors, without undue reservation.
