# Supplementary material for: Lactobacillus rhamnosus D3189 modulates antiviral and inflammatory responses in primary nasal epithelial cells, reducing respiratory syncytial virus shedding
Source: Front Cell Infect Microbiol. 2025 Jul 8;15:1625517. doi: 10.3389/fcimb.2025.1625517 (PMC12279880; doi:10.3389/fcimb.2025.1625517)
Supplement: Supplementary file 1 [file DataSheet1.pdf]

Supplementary Material

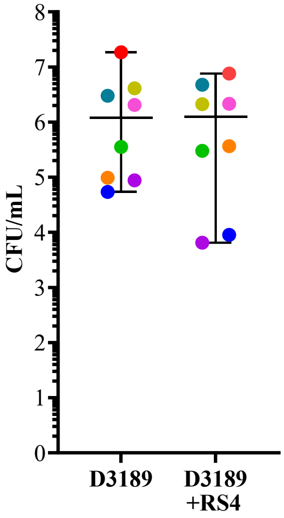

Supplementary Figure 1. *L. rhamnosus* D3189 load at 4 days post-exposure. WD-NECs were pretreated with D3189 (2.0 x 10^6 CFU in 80 µL) or media (negative control) for 24 h before RSV infection (6.0 x 10^5 PFU in 80 µL) or mock exposure (virus-negative control). At 3 days post-infection, apical washes were collected, and the drop plate technique was used to quantify the live bacteria on the apical surface. No bacteria were recovered from the cultures exposed to media. Data are presented as median and interquartile range. Each data point represents the mean of duplicate cultures per donor, with donors distinguished by colour (n = 8).

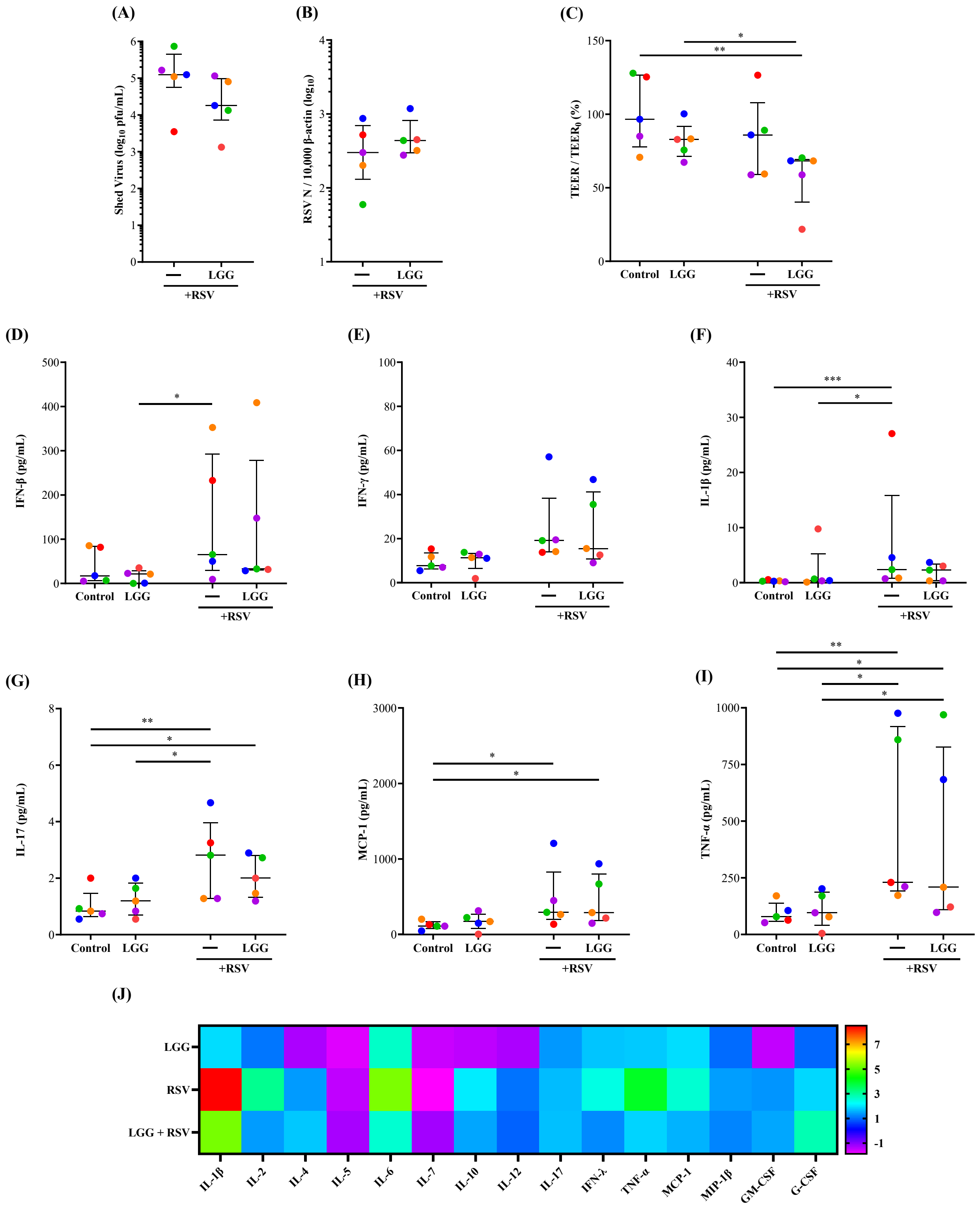
(A)
Shed Virus (log10 pfu/mL)
LGG
+RSV
(B)
RSV N / 10,000 β-actin (log10)
LGG
+RSV
(C)
TEER / TEER0 (%)
Control
LGG
LGG
+RSV
(D)
IFN-β (pg/mL)
(E)
IFN-γ (pg/mL)
(F)
IL-1β (pg/mL)
(G)
IL-17 (pg/mL)
(H)
MCP-1 (pg/mL)
(I)
TNF-α (pg/mL)
(J)
LGG
RSV
LGG + RSV
IL-1β
IL-2
IL-4
IL-5
IL-6
IL-7
IL-10
IL-12
IL-17
IFN-λ
TNF-α
MCP-1
MIP-1β
GM-CSF
G-CSF

Supplementary Figure 2. Effect of *L. rhamnosus* LGG on viral load, TEER, and the immune response 3 days post-infection with RSV. WD-NECs were pretreated with LGG (80 µL of 2.5×10^7 CFU mL^{-1}) or media (negative control) for 24 h before RSV infection (80 µL of 7.5×10^6 PFU mL^{-1}) or mock exposure (virus-negative control). At 3 days post-infection, apical washes and basal media were collected. **(A)** Shed virus was quantified by immuno-plaque assay using apical washes. **(B)** Viral transcription was assessed by RT-qPCR using RSV N-specific primers, normalised to β-actin expression. **(C)** TEER was measured using the $EVOM^2$ Epithelial Voltohmmeter and is presented as a percentage fold change relative to baseline TEER. **(D)** IFN-β was quantified by AlphaLISA using both apical wash and basal media. Secreted pro-inflammatory cytokines and chemokines were measured in basal media using the Bio-plex pro human cytokine 17-plex assay. Data points represent secreted **(E)** IFN-γ, **(F)** IL-1β, **(G)** IL-17, **(H)** MCP-1, and **(I)** TNF-α from each donor. A **(J)** heatmap illustrates fold changes in cytokine and chemokine production relative to uninfected controls for all analytes detected with the assay's limit of detection. Data are presented as median and interquartile ranges, analysed using either the Wilcoxon signed-rank test or the Friedman test with uncorrected Dunn's post hoc test. Each data point represents the mean of duplicate cultures per donor, with donors distinguished by colour ($n = 5$). *, $p < 0.05$; **, $p < 0.01$; ***, $p < 0.005$.
